# Supplementary material for: Effect of pre-operative bicarbonate infusion on maternal and perinatal outcomes among women with obstructed labour in Mbale hospital: A double blind randomized controlled trial
Source: PLoS One. 2021 Feb 9;16(2):e0245989. doi: 10.1371/journal.pone.0245989 (PMC7872290; doi:10.1371/journal.pone.0245989)
Supplement: S2 File — (DOCX) [file pone.0245989.s003.docx]

Subgroup analysis;

In a prespecified sub-group analysis, there were substantial differences in maternal venous blood lactate at one hour between the intervention and control groups among none referred participants with OL [( median difference 3 mmol/L;p-value=0.009: Vergha and Delaney effect size (A) was 0.39 (0.30 – 0.47)] and those that gave birth within 120 minutes of receiving the intervention [( median difference 1.5; p-value 0.020: Vergha and Delaney effect size (A) was 0.42 (0.35 – 0.49)]. Details of the outcomes are in tables 5a and 5b.

Table 5a; Subgroup analysis by history of being referred from a lower health facility with OL.

| **Subgroup** | **Outcome** | **Lactate: Median (IQR)** | | **Abs diff in Medians** | **Effect size VDA****  **(95% CI)** | **P-value*** |
| --- | --- | --- | --- | --- | --- | --- |
|  |  | **Intervention (n=238)** | **Control (n=239)** |  |  |  |
| **Referred (n=303)** | **Maternal venous blood lactate, mmol/L** | | | | | |
|  | At baseline | 6.5 (3.2,13.9) | 7.3 (3.7, 12.8) | 0.8 | 0.48 (0.42,0.54) | 0.550 |
|  | At 1 hour | 6.5 (3.3,12.6) | 6.8 (3.5,13.7) | 0.3 | 0.50 (0.43,0.57) | 0.955 |
|  | Myometrial blood | 4.1 (2.9,7.4) | 4.8 (3.3,6.7) | 0.7 | 0.39 (0.32,0.45) | 0.203 |
|  | **Fetal cord blood lactate, mmol/L** | | | | | |
|  | Artery | 9.1 (5.2,14.8) | 9.1 (5.7,14.7) | 0 | 0.49 (0.42,0.55) | 0.659 |
|  | Vein | 7.5 (4.8,12.0) | 9.0 (5.3,13.1) | 1.5 | 0.45 (0.38,0.51) | 0.112 |
|  | **Perinatal condition** | **(n=160)** | **(n=143)** |  | | |
|  | Alive | 133 (83.2) | 125 (87.4) | - | - | 0.514*** |
|  | Dead | 19 (11.9) | 14 (9.8) |  |  |  |
|  | Missing | 8 (5.0) | 4 (2.8) |  |  |  |
| **Not referred (n=174)** | **Maternal venous blood lactate, mmol/L** | | | | | |
|  | At 0 baseline | 6.6 (3.2,11.0) | 7.4 (3.7,12.3) | 0.8 | 0.46 (0.38,0.55) | 0.384 |
|  | At 1 hour | 5.8 (3.3,10.7) | 8.8 (5.1,21.2) | 3 | 0.39 (0.30,0.47) | 0.009 |
|  | Myometrial blood | 4.6 (3.3,6.2) | 4.8 (3.4,8.1) | 0.6 | 0.38 (0.3,0.46) | 0.188 |
|  | **Fetal cord blood lactate, mmol/L** | | | | | |
|  | Artery | 7.6 (5.3,12.4) | 8.2 (5.2,12.5) | 1.2 | 0.50 (0.41,0.58) | 0.940 |
|  | Vein | 6.6 (4.7,12.3) | 6.5 (4.2,11.1) | -0.1 | 0.53 (0.45,0.62) | 0.489 |
|  | **Perinatal condition** | **(n=78)** | **(n=96)** |  | | |
|  | Alive | 77 (98.7) | 90 (93.8) | - | - | 0.154*** |
|  | Dead | 1 (1.3) | 5 (5.2) |  |  |  |
|  | Missing | 0 (0) | 1 (1.0) |  |  |  |

* Mann-Whitney U test; ** VDA is Vargha and Dalaney's A; Abs. diff. is Absolute difference, *** Frequencies (%) with Fischer’s exact test.

Table 5b; Subgroup analysis by duration in minutes from drug administration to childbirth.

| **Subgroup** | **Outcome** | **Lactate: Median (IQR)** | | **Abs diff in Medians** | **Effect size VDA*****  **(95% CI)** | **P-value*** |
| --- | --- | --- | --- | --- | --- | --- |
|  |  | **Intervention (n=238)** | **Control (n=239)** |  |  |  |
| **Less than 120 min (n=295)** | **Maternal venous blood lactate, mmol/L** | | | | | |
|  | At baseline | 7.4 (3.8,15.6) | 8.0 (4.2,13.9) | 0.6 | 0.49 (0.42, 0.55) | 0.656 |
|  | At 1 hour | 6.1 (3.3,11.8) | 7.6 (4.2,17.6) | 1.5 | 0.42 (0.35, 0.49) | 0.020 |
|  | Myometrial blood | 4.8 (3.4, 6.9) | 4.6 (3.3,7.2) | - 0.2 | 0.45 (0.38, 0.51) | 0.907 |
|  | **Fetal cord blood lactate, mmol/L** | | | | | |
|  | Artery | 9.1 (5.6,14.1) | 8.2 (4.9,13.3) | -0.9 | 0.53 (0.46, 0.60) | 0.442 |
|  | Vein | 7.7 (5.0,12.2) | 7.2 (4.2, 11.9) | -0.5 | 0.53 (0.47, 0.60) | 0.367 |
|  | **Perinatal condition** | **(n=142)** | **(n=153)** |  | | |
|  | Alive | 123 (86.6) | 138 (90.2) | - | - | 0.641*** |
|  | Dead | 13 (9.2) | 12 (7.8) |  |  |  |
|  | Missing | 6 (4.2) | 3 (2.0) |  |  |  |
| **More than 120 minutes (n=182)** | **Maternal venous blood lactate, mmol/L** | | | | | |
|  | At 0 baseline | 4.8 (2.6,10.4) | 5.8 (3.3,10.2) | 1.0 | 0.46 (0.38,0.54) | 0.356 |
|  | At 1 hour | 6.9 (3.7,14.2) | 7.2 (3.93, 13.5) | 0.3 | 0.51 (0.42, 0.60) | 0.841 |
|  | Myometrial blood | 3.8 (2.7,6.5) | 5.5 (3.3, 8.3) | 1.7 | 0.29 (0.22,0.38) | 0.004 |
|  | **Fetal cord blood lactate, mmol/L** | | | | | |
|  | Artery | 7.7 (5.1,13.0) | 9.35 (6.2,13.7) | 1.7 | 0.43 (0.35,0.52) | 0.126 |
|  | Vein | 6.6 (4.5,12.0) | 8.90 (6.15,13.3) | 2.4 | 0.40 (0.32,0.49) | 0.019 |
|  | **Perinatal condition** | **(n=96)** | **(n=86)** |  | | |
|  | Alive | 87 (90.1) | 77 (89.5) | - | - | 0.826*** |
|  | Dead | 7 (7.3) | 7 (8.1) |  |  |  |
|  |  | 2 (2.1) | 2 (2.3) |  |  |  |

* Mann-Whitney U test; ** VDA is Vargha and Dalaney's A; Abs. diff. is Absolute difference, *** Frequencies (%) with Fischer’s exact test.
