# Supplementary material for: Effect of pre-operative bicarbonate infusion on maternal and perinatal outcomes among women with obstructed labour in Mbale hospital: A double blind randomized controlled trial
Source: PLoS One. 2021 Feb 9;16(2):e0245989. doi: 10.1371/journal.pone.0245989 (PMC7872290; doi:10.1371/journal.pone.0245989)
Supplement: Attachment — Submitted filename: Response to further editor comment.doc [file pone.0245989.s009.doc]

29^th^ /12/2020

To

Dr. Jacqueline J. Ho

Academic Editor

PLOS ONE

Dear Dr. Jacqueline J. Ho,

**Re; Response to reviewers’ comments and resubmission of revised manuscript PONE-D-20-25151R3**
Effect of pre-operative bicarbonate infusion on maternal and perinatal outcomes among women with obstructed labour in Mbale hospital: a double blind randomized controlled trial.

Thank you for reviewing and providing feedback on this manuscript. Please receive the revised copy with specific responses and changes summarized in the table below.

| **Reviewers comment** | **Response to comment** | **Line number** |
| --- | --- | --- |
| **Academic editor** | | |
| Thank you for revising Table 1 as requested. Do you think it would be better like this? | Yes, we agree. It looks alright | Page 9 - 0  Lines 278 - 281 |

END
